# Printed Multilayer Piezoelectric Transducers on Paper for Haptic Feedback and Dual Touch-Sound Sensation

**DOI:** 10.3390/s22103796

**Published:** 2022-05-17

**Authors:** Georg C. Schmidt, Jonas M. Werner, Thomas Weissbach, Jörg Strutwolf, Robert Eland, Welf-Guntram Drossel, Arved C. Hübler

**Affiliations:** 1 Institute for Print and Media Technology, Chemnitz University of Technology, 09126 Chemnitz, Germany; thomas.weissbach@mb.tu-chemnitz.de (T.W.); joerg.strutwolf@mb.tu-chemnitz.de (J.S.); zaphod_beeblebrox@systemli.org (R.E.); arved.huebler@mb.tu-chemnitz.de (A.C.H.); 2 Professorship for Adaptronics and Lightweight Design in Production, Chemnitz University of Technology, 09126 Chemnitz, Germany; jonas-maximilian.werner@mb.tu-chemnitz.de (J.M.W.); welf-guntram.drossel@mb.tu-chemnitz.de (W.-G.D.); 3 Fraunhofer Institute for Machine Tools and Forming Technology, 09126 Chemnitz, Germany

**Keywords:** printed actuators, paper electronics, screen printing, haptics, piezoelectrics, P(VDF-TrFE)

## Abstract

With a growing number of electronic devices surrounding our daily life, it becomes increasingly important to create solutions for clear and simple communication and interaction at the human machine interface (HMI). Haptic feedback solutions play an important role as they give a clear direct link and response to the user. This work demonstrates multifunctional haptic feedback devices based on fully printed piezoelectric transducers realized with functional polymers on thin paper substrate. The devices are flexible; lightweight and show very high out-of-plane deflection of 213 µm at a moderate driving voltage of 50 V_rms_ (root mean square) achieved by an innovative multilayer design with up to five individually controllable active layers. The device creates a very clear haptic sensation to the human skin with a blocking force of 0.6 N at the resonance frequency of 320 Hz, which is located in the most sensitive range of the human fingertip. Additionally the transducer generates audible information above two kilohertz with a remarkable high sound pressure level. Thus the paper-based approach can be used for interactive displays in combination with touch sensation; sound and color prints. The work gives insights into the manufacturing process; the electrical characteristics; and an in-depth analysis of the 3D deflection of the device under variable conditions

## 1. Introduction

Our daily life is more and more surrounded and influenced by complex, multifunctional electronic devices. These devices are often controlled by human machine interfaces (HMI) giving options for input and output signals. Commonly, visual and acoustical feedback is used. An active tactile feedback to create additional or alternative haptic sensations at the point of interaction has rarely been incorporated into electronic devices so far. However, there has been a growing interest in the field of haptics over the last decade, mainly driven by the wide market penetration of touch screens and the latest developments in virtual environments and wearables [1,2].

We can appreciate a sense of touch by the use of device-generated environments; these are referred to as haptic devices. As a result, virtual objects appear real and tangible when touching them. It is possible for the user to create an interface with a virtual environment using the haptic technology; this is achieved through the touching sense, making use of forces, vibrations, or motions available to the user. Such mechanical simulation contributes to the creation of virtual objects, the control of virtual objects, and augmentation of the remote control properties of different machines and devices [3].

There have been numerous attempts to understand and apply haptic abilities for both humans and machines. This can be seen in the large number of activities in disciplines like robotics and telerobotics, computational geometry and computer graphics, psychophysics, cognitive sciences, and neurosciences [4]. Additionally, haptic devices can find application in more general areas that can be related to the virtual environments; such as medicine [5], gaming [6,7], robotics [8], communication [9], mobile devices [10], data visualization and multi-user environments [11].

In particular, polymers are very attractive for the realization of flexible electronics, e.g., to be used as electronic skin, interface for HMIs, physiological signal monitoring, and so on [12].

In this case, piezoelectric polymer actuators are devices made of smart materials capable of undergoing deformations in response to suitable electrical stimuli. They represent an emerging class of electromechanical drivers. Piezoelectric polymer actuators show functional and structural properties that have no equals among traditional actuation technologies (namely electrostatic and electromagnetic), such as large active strains in response to driving voltages, high power density, high mechanical compliance, structural simplicity and versatility, scalability, no acoustic noise, and, in most cases, low costs [13,14,15]. Polyvinylidene fluoride (PVDF) and its co- and ter-polymers can be seen as one of the best candidates for mechanical and acoustic sensors, actuators, energy harvesters and non-volatile memory applications [16]. Novel inorganic ferroelectrics, like lead-free Bi_0.5_Na_0.5_TiO_3_ (BNT) and antimony sulfoiodide nanowires, also show remarkable piezoelectric properties. However, they show limitations for simple processing technologies and require typically high sintering temperature not compatible to most flexible substrate materials [17,18].

With respect to haptic applications, Ju et al. demonstrated tactile feedback on flexible touch screens; accordingly, they designed and fabricated transparent relaxor ferroelectric polymer film vibrators with solution processed but non-printed poly(vinylidene fluoride-trifluoroethylene-chlorotrifluoroethylene) (P(VDF-TrFE-CTFE)) as the active layer. This tactile-feedback touch screen had a natural frequency designed to be around 200–240 Hz close to the highest sensitivity range of the human fingertips, which is around 300 Hz as reported by Gescheider et al. [19]. Thus, in a laminate structure combining touch sensors, the film vibrator and a flexible display panel, an advanced user experience can be created [20].

For a cost-effective production and the realization of large-area flexible actuators, the combination of organic actuator materials and additive printing technologies can play an important role. Poncet et al. presented the study of screen printed P(VDF-TrFE) based haptic circular buttons, providing force restitution or vibration sensations when touched by the user causing a tactile sensation on the human fingertips [21]. The first resonant mode frequency of an 18 mm diameter membrane was simulated to be 1.937 kHz with a deflection of 1.5 µm at 6 V_rms_. Digital inkjet printing was the method selected for the manufacturing of piezoelectric P(VDF-TrFE) layers [22].

Our group demonstrated fully mass printed large-area piezoelectric actuators on the basis of electroactive polymers (EAP) on thin foil and paper substrates for flexible loudspeakers [18,19,20,21]. Recently, a truly fully roll-to-roll manufacturing line for paper embedded transducers was presented [23].

In this report, the low-cost mass printing approach was used as a manufacturing route for powerful large area multilayer piezoelectric actuators for haptic sensations. For the first time, haptic feedback devices on environmentally friendly flexible and lightweight paper substrate with individually controllable multilayer architecture are demonstrated to show an extremely high deflection of up to 213 µm at a resonance frequency of 320 Hz, perfectly fitting to the highest fingertip sensitivity. This novel approach generates a blocking force of 0.6 N for the haptic feedback, which is sufficient to generate an indentation on the human skin. To improve the user sensation, the haptic feedback can be combined with audible sound information as the transducers show a high sound pressure level (SPL) above two kilohertz. To complete the multifunctional approach, and to be able to combine feedback and perception, the device can also be used as a touch sensor.

## 2. Materials and Methods

### 2.1. Device Set-Up and Printing

The printed piezoelectric transducers were manufactured in a similar way as already described previously [24]. However, for the multilayer approach of this work, the printing design and the sequence of printing was changed. In a brief summary: the basis of the transducers is a conventional, glossy coated paper substrate with a thickness of 67 µm and a grammage of 90 g m^−2^ (Maxigloss, IGEPA group GmbH & Co. KG, Hamburg, Germany). The device set-up included a layer sequence with up to six electrodes and up to five piezoelectric layers printed alternately one upon the other. While the electrodes were made of a water-based poly(3,4-ethylenedioxythiophene) polystyrene sulfonate (PEDOT:PSS) ink (Clevios SV4, Heraeus Deutschland GmbH & Co. KG, Hanau, Germany) resulting in a dry layer thickness of ~500 nm each. P(VDF-TrFE) (75:25 mol%) (FC25, Piezotech Arkema, Pierre-Benite Cedex, France) powder was dissolved in a high-boiling point solvent to prepare a screen printing compatible ink with good film-levelling properties. Depending on the target layer thickness (~7 µm), the co-polymer concentration of the ink can be varied between 15 and 20%wt. The PVDF-co-polymer was selected as it creates much higher deflection at a comparable low electric field strength below 20 V µm^−1^ compared to ter-polymers, which is important for usability [25].

**Figure 1:**
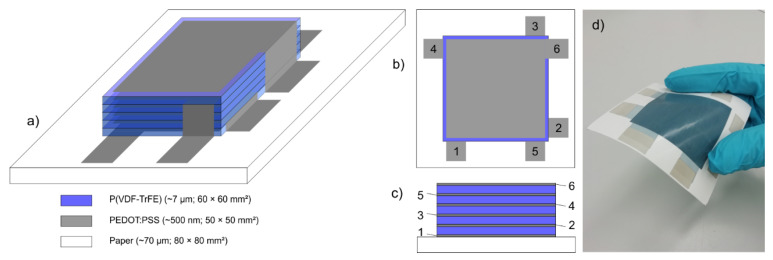
Multilayer device set-up: (**a**) 3D schema; (**b**) top view with electrode numbers on contact pads; (**c**) cross sectional view of the device with six electrodes and five active P(VDF-TrFE) layers; (**d**) Photo of the printed paper based transducer.

In contrast to other publications on printed piezoelectric multilayer devices [26,27], the layer design offers the opportunity for individual electrical connection and contacting of each electrode layer. To achieve this, the design included contact pads (15 × 15 mm^2^), which are shorter than the edge length of the quadratic electrode areas (50 × 50 mm^2^) themselves. Furthermore, the design is rotationally symmetric, which gives the possibility of printing all electrodes for transducers with up to five active layers with two printing forms only. The design of all piezoelectric layers was identical with a size of 60 × 60 mm^2^ providing tolerance against misalignment errors and safety against shorts of the electrodes especially at the edges. A schematic illustration of the design and the layer configuration of the multilayer device is shown in Figure 1.

All contact pads were supported with respect to their electrical conductivity by printing a silver ink (Dupont 5028, Dupont Ltd., Bristol, U.K.) on top of the conductive polymer. The thickness of the silver pads was measured to be ~5 µm.

All printing steps were carried out at the semi-automatic sheetfed screen printing press Ekra X1-SL (Ekra Automatisierungssysteme GmbH, Bönnigheim, Germany) with video assisted but manual alignment of the samples before each printing step. After each printing step, the layers were dried in a hot-air convection oven. The drying conditions for PEDOT:PSS, P(VDF-TrFE) and silver ink were 130 °C/5 min, 135 °C/10 min and 130 °C/5 min, respectively.

### 2.2. Poling

**Figure 2:**
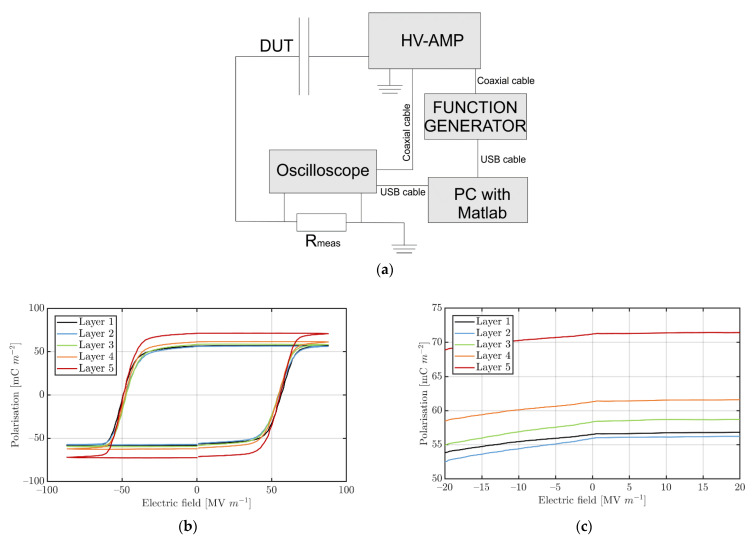
(**a**) Schema of the poling set-up. P-E-Loops of (**b**) each individual layer within a multilayer stack device with five active P(VDF-TrFE) layers. (**c**) Detail of the P-E-Loops showing the layer-to-layer difference in remanent polarisation more precisely.

The crucial advantage of the developed design is the possibility to control each active layer individually. This includes the poling procedure, which is necessary to get a high remnant polarization of the P(VDF-TrFE) layer. Poling was conducted with the help of a so called Sawyer-Tower circuit by applying a high-voltage (HV) signal with triangular waveform and a frequency of 1 Hz. For this, samples were connected with a HV supply (Trek 5/80-HS, Acal BFi Germany GmbH, Dietzenbach, Germany), which was controlled by using a function generator (TGA1244, Telemeter Electronic GmbH, Donauwörth, Germany) and an HV resistor of 987 Ω. The current flowing through the circuit was determined by measuring the voltage of the resistor using an oscilloscope (DSO-X 2004A, Agilent Technologies Inc., Santa Clara, CA, U.S.A.) via a 100:1 probe (10076B, Agilent). The poling set-up is depicted in Figure 2a.

More precisely, two positive cycles were followed by two negative cycles, which allow the polarization–electric field (P-E)-hysteresis loop to be calculated by measuring the total current during the first cycle, including the parasitic parts (leakage and charging currents) and subtracting them with the help of the current measurement (only the parasitic current remains) during the second cycle. A maximum electric field of 100 V µm^−1^ was applied to the individual layers, which is much higher than the coercive field and a typical value to achieve the high remnant polarization of printed P(VDF-TrFE) layers. It is important to mention that the stepwise individual polarization of single layers strongly reduces the risk of electrical breakdowns damaging the devices in contrast to the parallel polarization of all active layers within a standard multilayer device.

### 2.3. D-Vibrometry

To determine the vibration mode and the frequency of the first resonance frequency, non-contact scanning measurements using a Scanning Vibrometer (PSV-500-3D, Polytec GmbH, Waldbronn, Germany) were carried out. The expanded uncertainty for the measurements is 3% as stated by the manufacturer.

For this, a swept sine wave ranging from 100 Hz to 1000 Hz with 1 s in length and an amplitude of 0.2 V was produced by the integrated waveform generator of the measurement system. The signal was amplified with a factor of 70.7 to achieve a voltage with an RMS value of 10 V_rms_. The resulting surface displacement was measured using the laser scanning vibrometer system with a bandwidth of 4 kHz. Using three scanning heads, the vibration was investigated in all three spatial directions for a large part of the active area of the sample (approx. 36 × 40 mm^2^).

For the displacement measurements, the same set-up was used. However, a fixed sinusoidal signal with the resonant frequency and a voltage of 10–50 V_rms_ was applied. The individual layers are connected in parallel for all experiments.

## 3. Results and Discussion

To better evaluate the electrical and mechanical properties of the actuator, different experiments are carried out. Firstly, the polarization of each individual layer is measured to show the differences between them. Furthermore, the capacitance of each layer, and also the complete multilayer device as well as the dissipation factor, is measured. Secondly, for the mechanical properties, the resonance frequency of the actuator is determined and measurements for different driving voltages and a varying number of active layers are carried out. Lastly, to get a better understanding for the use of the printed multi-layered actuator as a haptic feedback device, experiments to determine the blocking force of the actuator are conducted.

### 3.1. Poling and Dielectric Spectroscopy

After finalizing all printing and annealing steps, all manufactured devices were polarized layer by layer to achieve high piezoelectricity of the P(VDF-TrFE) films. High remnant polarization of the piezoelectric layers is an important prerequisite to achieve high deflection of the actuator [24]. Figure 2b shows the individual P-E-loops of printed multilayer devices with five active layers. Interestingly, there is a clear trend visible from the top layer (Figure 2c, Layer 5) to the bottom (Layer 1). This means, the individual P_r_ values from the fifth layer (P_r_~72 mC m^−2^) to the first layer decreases. In particular, between the top layer and the fourth layer, there is a large decrease in P_r_ by ~10 mC m^−2^. The further decrease is comparably small; from layer 4 to layer 1 the decrease was measured to be ~4 mC m^−2^. This result occurred for all tested multilayer samples independent of the number of functional layers. For single layer devices, the highest remnant polarization was measured, which can be explained by the single active layer also bring the top layer in this case.

It is well known that the crystallinity of P(VDF-TrFE) films is highly temperature sensitive [28]. To achieve high crystallinity, careful annealing slightly above the Curie temperature of the solution processed layers is necessary. For the selected co-polymer with a PVDF:TrFE ratio of 75:25, an annealing temperature of 135 °C is recommended [29]. However, the annealing temperature was the same for all samples prepared for this study. Further influence could come from differences in the total annealing time and the number of annealing cycles as multilayer devices were annealed as often as the number of printed active layers in addition to the number of printed electrodes. However, to the best of our knowledge, such a clear difference has not been published before. Hence, further investigations, especially with respect to the morphology of the involved layers, should be carried out in succeeding studies to achieve a maximized remnant polarization throughout all active layers, which would improve the overall performance of the multilayer actuator.

**Figure 3:**
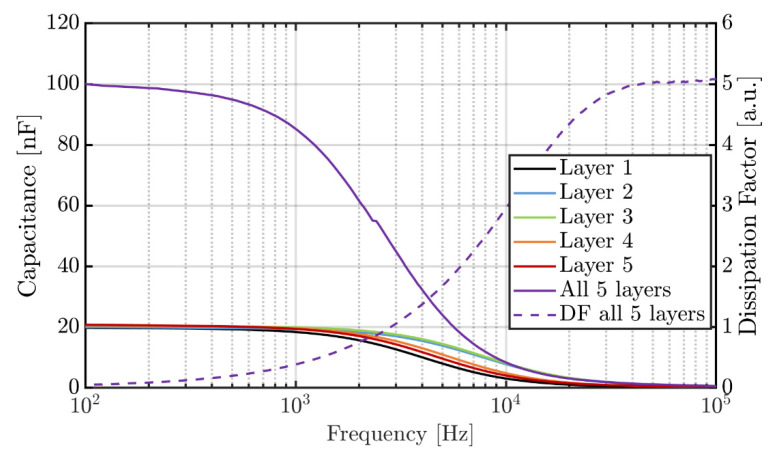
Capacitance and dissipation factor of individual layers and multilayer device.

The frequency spectra of the capacitance after poling of all individual layers and the parallel connection of them is shown in Figure 3.

The small deviation of the curves clearly indicates the high layer-to-layer homogeneity with respect to the thickness and dielectric properties of the individual P(VDF-TrFE) layers and the conductivity of the electrodes, which is important to achieve highly efficient superposition of the forces generated by each individual active layer [24]. The decrease in capacitance for frequencies above ~2000 Hz can be attributed to the comparably low conductivity of the polymeric PEDOT:PSS electrodes as explained in detail in our former publications [30,31]. As the first electrode layer is printed directly on the paper surface, its conductivity is the lowest in comparison to the other layers. Hence, the decrease in capacitance occurs earliest for this layer. Basically, it should be noticed that the influence of this decrease is small for applications in the low frequency range like for the proposed haptic feedback and touch sensation paper. For the determined resonance frequency, a capacitance of 20.1 ± 0.4 nF per layer and a dissipation factor (*DF*) of 0.13 were measured. *DF* is defined as *DF* = tan(90° − *φ*) where *φ* is the phase shift between current and voltage.

### 3.2. Resonance Frequency and Deflection under Variable Conditions

**Figure 4:**
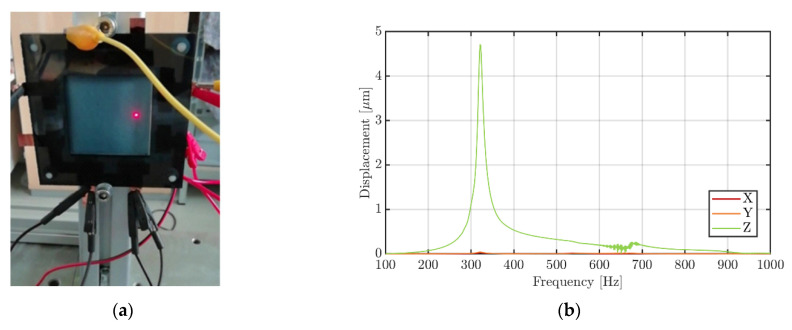
(**a**) Printed multilayer transducer mounted in a plastic frame during 3D laser vibrometer measurement; (**b**) Transfer function of the five-layer multilayer transducer.

The following section presents the results of the 3D vibrometer measurements to gain insight into the vibration behavior of a five-layer multilayer transducer as shown in Figure 1. At first, the resonance frequency of the device mounted and clamped between two stiff plastic frames (Figure 4a) was analyzed.

The transfer function of the resulting displacements in all three spatial directions when working with only one of the active layers (sine wave sweep signal with 50 V_rms_ applied to electrodes 1 and 2) is depicted in Figure 4b. The peak clearly shows the resonance frequency of the transducer system at approx. 320 Hz. Between the *z*-axis (out of plane) and the *x*- and *y*-axis, the difference in amplitude is 47 dB and 42 dB, respectively, which indicates a clear dominance of the vibration in the intended direction. The measured value is in good accordance with our own analytical calculations based on the following equations provided by Ju et al. [20] for the natural frequency *ω_n_* of multilayer piezoelectric transducers with *N* piezoelectric layers.

(1) $$\omega_{n}=\left(\beta_{n}\right)2\sqrt{\frac{{\left(YI\right)}_{eff}}{\left(\rho h_{eff}\right)}}$$

(2) $${\left(\rho h\right)}_{eff}=\overset{2N+3}{\underset{i=1}{\sum }}\rho_{i}h_{i}$$

With ${\left(YI\right)}_{eff}$ and ${\left(\rho h\right)}_{eff}$ being the effective flexural modulus per unit length and the effective weight per area, respectively. *ρ_i_* and *h_i_* represent the density and thickness of the *i*-th layer. *β_n_* is the eigenvalue of the *n*-th vibration mode determined by the boundary condition.

For the realized device geometry and taking 5.7 GPa, 3.6 GPa [32] and 1.0 GPa [20] as the Young modulus and 1389 kg m^−3^, 1800 kg m^−3^ and 1011 kg m^−3^ as the density for the used paper, the P(VDF-TrFE) layers, and the PEDOT:PSS layers, respectively, a natural frequency for the first natural mode of 344 Hz was calculated. Moreover, the Young modulus of paper is highly anisotropic due to the direction of the paper fibers [30], which was not considered in the calculations. As expected, no clear change of the resonance frequency in relation to the number of driven piezoelectric layers was observed.

In the following paragraph, the results of the analysis of the vibration measurements of such a multilayer piezoelectric transducer with five individually controllable active layers driven at the determined resonance frequency are given. The aim of the experiments was to investigate the influence of the applied voltage and the number of excited layers on the displacement achievable with the sample. For a better understanding of the influence of the voltage, the values varied from 10 V_rms_ to 50 V_rms_ in 10 V increments. Additionally, the measurements were repeated for 1–5 controlled active layers.

**Figure 5:**
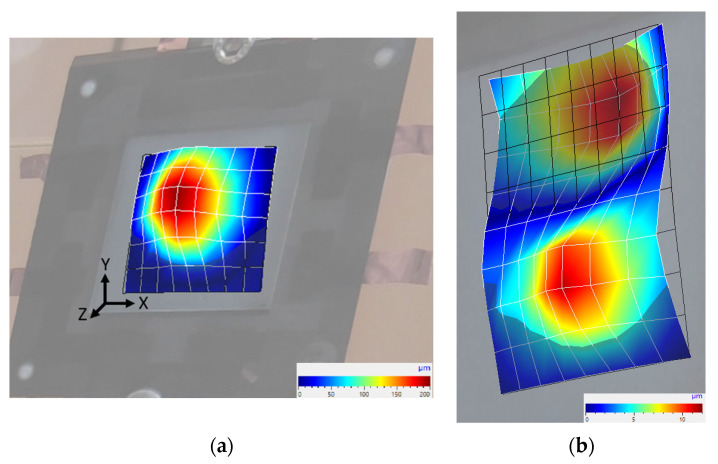
Deflection shape of the multilayer sample at: (**a**) 320 Hz (resonance frequency) and; (**b**) 640 Hz (second harmonic) at 50 V_rms_. All five P(VDF-TrFE) layers are parallel connected and activated.

The resulting deflection shapes of the first (320 Hz) and second harmonic (640 Hz) oscillation of the sample excited at the resonance frequency are shown in Figure 5. The (1,1) mode in the z-direction, centered on the middle of the sample, is clearly visible for the first harmonic oscillation. For the second harmonic oscillation, the (1,2) mode with one node is present. Its amplitude is strongly reduced, which is important to keep the harmonic distortion low. The vibration in the *x*- and *y*-direction is negligible and no vibration at the edges of the sample was observed due to the plastic frame used for clamping the sample. It could be confirmed that the expected mode shape for a fully clamped square film occurs for our paper based design as well. The deflection shape did not change for different voltages or different numbers of parallel connected excited layers.

**Figure 6:**
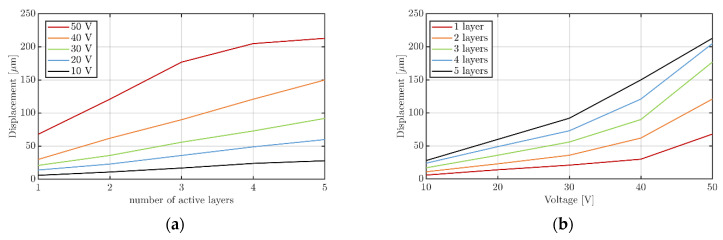
Resulting out-of-plane displacements in dependency of: (**a**) the number of active layers and; (**b**) the applied voltage.

Figure 6 shows the resulting out-of-plane peak displacement in dependency of the number of active layers and the driving voltage at a frequency of 320 Hz.

The values are summarized in Table 1. The highest achieved vibration amplitude is 213 µm, which corresponds to an acceleration of 861 m/s^2^. A near linear relation between voltage and the number of active layers can be detected at least for a driving voltage of up to 40 V_rms_. The incremental slope of the curves for 10 V_rms_ to 40 V_rms_ ranges from 0.55 to 0.78 µm V^−1^ per layer. The slope is only higher for the excitation with 50 V_rms_, than for the other voltages with 0.85 to 1.36 µm V^−1^ per layer. For 50 V_rms_ a saturation of the resulting displacement seems to start in the case of four and five driven piezoelectric layers, probably due to the limited device elasticity. There is also a voltage linearity of the displacement present up to a voltage level of 30 to 40 V_rms_, as shown in Figure 6b. For higher voltages, the displacement does not follow the linear increase anymore and a stronger increase becomes obvious. Such a behavior was already visible in the work of Ju et al., though at a much smaller displacement level [20].

To the best of our knowledge, the achieved out-of-plane displacement of more than 200 µm, at a supply voltage of 50 V_rms_, is the highest reported for polymer based piezoelectric haptic actuators, which is the result of the combination of a high performance of the printed layers, especially the piezoelectric one, the geometry of the substrate, and the low thickness and mechanical properties of the paper substrate. Table 2 gives a comparison to the work of other research groups and a commercially available device [32].

**Table 2:** Comparison of different flexible polymer based piezoelectric actuators used for haptic applications.

Piezoelectric Material	Substrate	Size	Voltage [V_rms_]	Active Layers	Working Frequency [Hz]	Out-of-Plane Displacement [µm]	Ref.
P(VDF-TrFE-CTFE)	PET, 188 µm	65 × 40 mm²	50	1	220	2	[17]
P(VDF-TrFE-CTFE)	PET, 188 µm	65 × 40 mm²	35	2	220	3.5	[17]
P(VDF-TrFE)	PEN, 125 µm	Ø12 mm	35	1	3410	8	[32]
P(VDF-TrFE)	n.a.	14 × 19 mm² (incl. frame)	53	n.a.	150	95	[33]
P(VDF-TrFE)	paper, 67 µm	50 × 50 mm²	50	5	320	213	this work

### 3.3. Blocking Force

**Figure 7:**
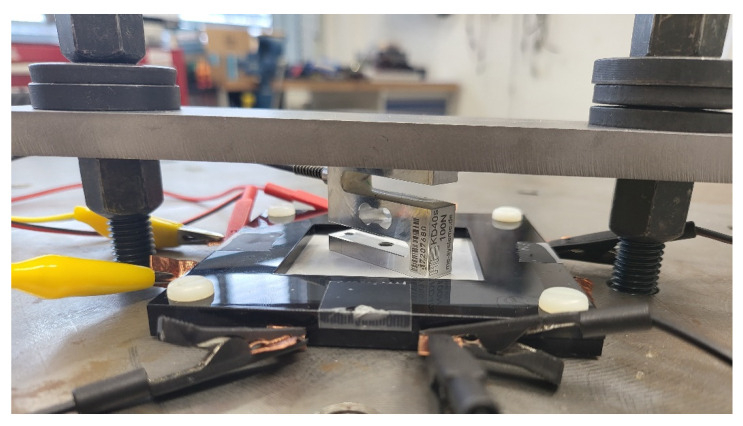
Measurement setup for the blocking force.

To use the transducer as a haptic device the generated blocking force is an important factor. To measure this, the displacement of the actuator needs to be completely blocked, i.e., it should work against a load with an infinitely high stiffness. To achieve this, the sample was mounted as shown in Figure 7. Under the sample, a square-shaped piece of poly(methyl methacrylate) (PMMA) was placed between the work surface and the sample. For the force measurement, a 1-axis force sensor (KD40s, ME-Meßsysteme GmbH, Hennigsdorf, Germany) with a nominal force of 100 N was placed on the sample. In order to ensure the high stiffness of the setup, the force sensor and the sample were preloaded with a steel plate, which was adjusted with screws. By doing this, a preload of 10 N was applied to the transducer. The testing rig used for measuring the blocking force is not optimal and should be optimized in future studies, but it should give an insight into the magnitude of the blocking force of the actuator.

**Figure 8:**
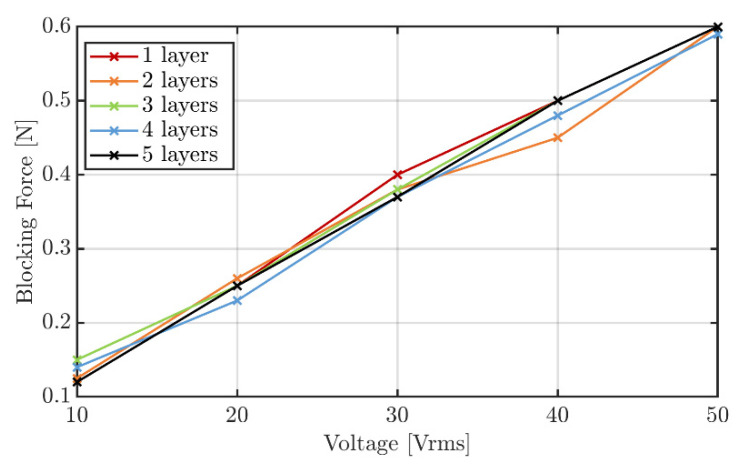
Blocking force of the transducer for different voltages and numbers of active layers.

The results show that the transducer generates a maximum blocking force of approx. 0.6 N at the resonance frequency of 320 Hz when using a voltage of 50 V_rms_. Linear behavior between the voltage and the generated blocking force is evident (Figure 8).

**Figure 9:**
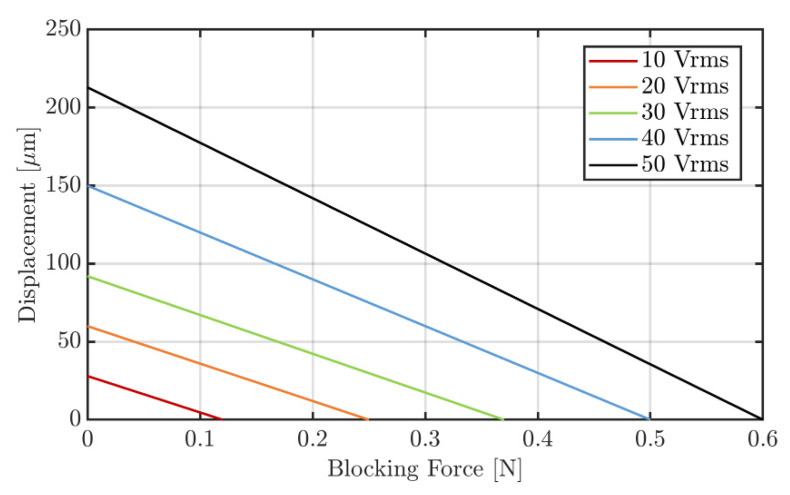
Working graph of the transducer for different voltages, all five layers were active. It should be noticed, the blocking force and the out-of-plane displacement of the transducer were measured at ambient conditions where small changes in temperature and relative humidity may have occurred.

Furthermore, as was to be expected, the number of active layers has no effect on the generated blocking force. The reason for this is, despite the fact that the individual layers are connected in parallel electrically, they are connected in a series mechanically, so the resulting blocking force is independent of the number of active layers. The corresponding working graph, when all five layers are active, is shown in Figure 9. The two important parameters of the transducer, the free stroke as well as the blocking force, are shown for different driving voltages as the values at the intersections with the *x*- and *y*-axis, respectively. During the operation of the transducer, any displacement/force point on and below the curves is attainable.

### 3.4. Acoustical Performance

**Figure 10:**
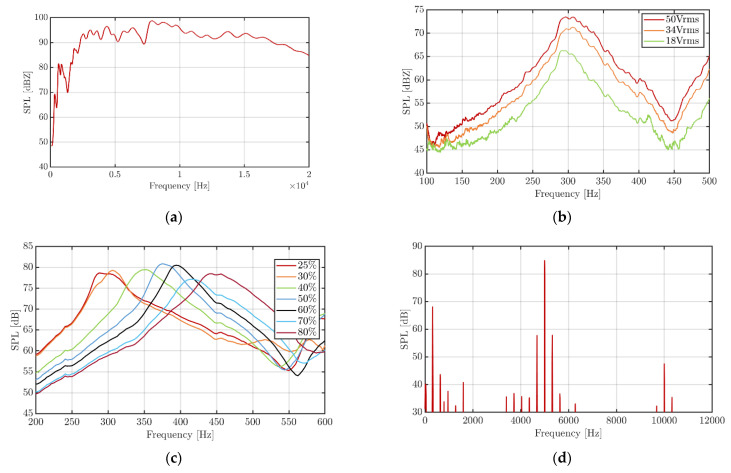
Acoustic performance of the printed multilayer transducer: (**a**) Frequency response for the full audio range from 100 Hz to 20 kHz; (**b**) Voltage and; (**c**) humidity dependency of the SPL for the haptic feedback relevant frequency range; (**d**) Frequency spectrum of the SPL for an applied bitonal sinusoidal signal combining 320 Hz and five kilohertz.

In addition to the vibration measurements, acoustical sound pressure level (SPL) measurements were performed in the audible frequency range from 100 Hz to 20 kHz. Figure 10a shows the frequency response of a multilayer transducer driven by a sinusoidal signal with 50 V_rms_.

The SPL was recorded at a distance of 10 cm. It is worth mentioning that, despite the flat mounting of the device, which is in contrast to our former publications on printed piezoelectric loudspeakers [23,24,30,31,34] and the compact size of the transducer, a remarkably high SPL of more than 90 dB was achieved for a wide frequency range from ~2 kHz to 17 kHz. For the haptics application, the low frequency range between 100 and 500 Hz is important. Here the SPL is much lower, which is actually beneficial for the proposed application as the audible noise is comparably small. However, the resonant frequency is clearly detectable via the SPL frequency response.

The SPL increases with increased driving voltage, as shown in Figure 10b, as expected. It should be noticed that the SPL includes harmonic distortions produced by higher mode oscillations (see also Figure 5). For the resonant frequency, the total harmonic distortion was measured to be ~50%. This value is much higher than the total harmonic distortion measured for frequencies above 500 Hz for printed piezoelectric speakers on paper, which is typically below 1% [34].

Small differences in the resonance frequency between the results of the laser vibrometer and the SPL measurements are due to the non-uniformity of the ambient laboratory conditions. In particular, the hygroscopic nature of the paper substrate can result in changes of the resonance frequency as the water absorption depending on the humidity in the lab can influence the mechanical properties (e.g., the bending stiffness) of the paper. To get a clear image of this interesting effect, which is of importance for the addressed haptic application, the influence of the humidity on the resonance frequency and the SPL was evaluated in more detail. For this, the multilayer sample was placed in a climate chamber (KPK 200, Feutron Klimasimulation GmbH, Langenwetzendorf, Germany), keeping the temperature constant (23 °C) and varying the relative humidity between 25% and 80%. As can be seen in Figure 10c, the peak of the SPL indicating the resonance frequency shifted from ~290 Hz at 25% relative humidity to ~450 Hz at 80%. It is well-known that paper absorbs water effectively leading to a decrease in the Young Modulus. However, other material properties like the density and the geometry can be affected as well. Hence, further studies are required to explain this result in accordance with Equation (1). Future work should also incorporate water repelling coatings to avoid or reduce this effect and, e.g., by the influence of human moisture at the fingertip.

To increase the interaction with the user, an interesting approach could be the combination of tactile and audible sensation [35]. To test this, a bitonal signal was applied to the transducer combining 320 Hz and 5 kHz sinusoidal signals with an amplitude of 47 V_rms_. Figure 10d shows the Fast Fourier Transform (FFT) of the measured SPL. Clearly visible are the two first harmonics of the signals with SPLs of 68.1 dB and 84.9 dB at 320 Hz and five kilohertz, respectively. Hence, the five kilohertz signal is very dominant and appears quite loud, while the 320 Hz signal produces a clearly detectable vibration. A video demonstrating the dual effect is given as Supporting Information.

As the transducer can also be used as a touch sensor by utilizing the direct piezoelectric effect (see Supporting Information Figure S1), a multifunctional touch display button with only one single printed device can be created.

## 4. Conclusions

In summary, fully screen printed multilayer piezoelectric transducers on paper with individually controllable active layers were demonstrated. The individual behavior of the piezoelectric polarization and the dielectric properties of each layer were shown. Examples of promising application areas for such thin and flexible transducer include haptics and touch sensations. Thus, in-deep 3D vibration measurements were realized. The resonant frequency for our device design is in the range of the highest sensitivity of the human finger tips and the deflection is high enough to produce a strong sensation to the human skin at moderate voltage level due to the multilayer set-up. The generated blocking force of the transducer is suffice to cause a skin indentation and therefore a haptic sensation. Applying a bitonal signal combining the resonance frequency and an audio signal above two kilohertz creates a dual touch-sound sensation. Hence, the transducer can be used as an interactive touch button for flexible paper-based displays and HMIs. In view of using the technology for real-world applications, future work should deal with suitable encapsulation materials to reduce the influence of ambient conditions. Furthermore, HMIs typically consist of more than one touch button, hence sensor arrays are needed. For such arrays, the interaction of adjacent touch points on the flexible substrate, as well as the dependence of the lateral size on the resonance frequency, have to be analyzed in order to reduce coupling effects and to keep the good haptic properties, respectively.

## Figures and Tables

**Table 1:** The out of plane displacement for different driving voltages and the number of active layers at resonance frequency. Additionally, the incremental slope is given.

	Number of Layers				
	1	2	3	4	5
Voltage [V_rms_]	Displacement [µm]/Slope [µm V^−1^ Layer^−1^]				
10	6/0.6	11/0.55	17/0.57	24/0.6	28/0.56
20	14/0.7	23/0.58	36/0.6	49/0.61	60/0.6
30	21/0.7	36/0.6	56/0.62	73/0.61	92/0.61
40	30/0.75	62/0.78	90/0.75	121/0.76	150/0.75
50	68/1.36	121/1.21	177/1.18	205/1.03	213/0.85

## Data Availability

Not applicable.

## Supplementary Materials

The following supporting information can be downloaded at: https://www.mdpi.com/article/10.3390/s22103796/s1, Figure S1: Sensing response of the multilayer device, stimulated by a human fingertip.
