# Supplementary material for: Printed Multilayer Piezoelectric Transducers on Paper for Haptic Feedback and Dual Touch-Sound Sensation
Source: Sensors (Basel). 2022 May 17;22(10):3796. doi: 10.3390/s22103796 (PMC9147910; doi:10.3390/s22103796)
Supplement: Supplementary file 1 [file sensors-22-03796-s001.zip › sensors-1692933-supplementary.pdf]

sensors

Supporting Information

Printed Multilayer Piezoelectric Transducers on Paper for Haptic Feedback and Dual Touch–Sound Sensation

Georg C. Schmidt *, Jonas M. Werner, Thomas Weißbach, Jörg Strutwolf, Robert Eland, Welf-Guntram Drossel and Arved C. Hübler

The printed multilayer piezoelectric transducer used as a touch sensor.

To showcase the potential of using the demonstrated piezoelectric actuator as a sensor, Figure S1 shows the output behavior of such a multilayer device when simply touching and slightly pushing the surface of the paper-based device by a human fingertip. The output voltage was recorded with the help of an oscilloscope (DSO-X 2004A, Agilent). The device was clamped in the same configuration as for the vibration measurement. The peak of the sensing response was in the range of ~800 mV and −400 mV for a push and release sequence.

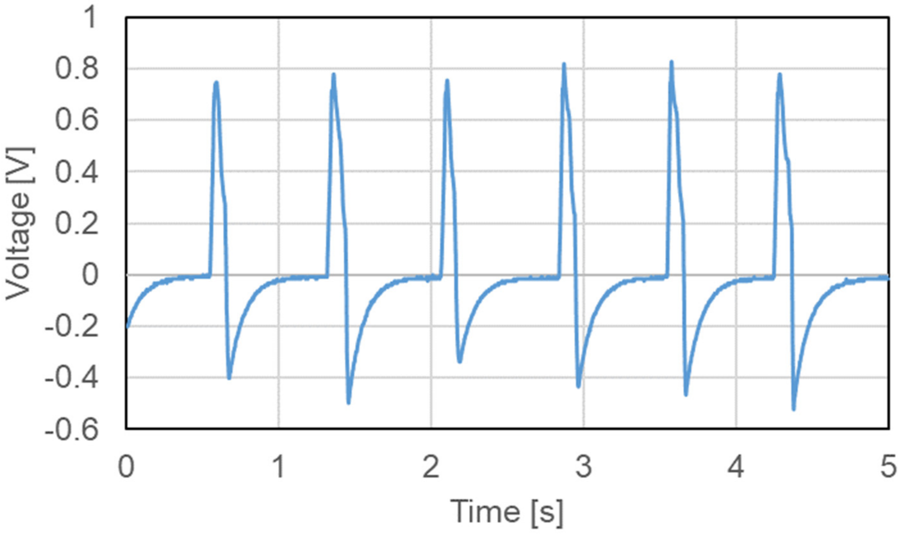

Figure S1. Sensing response of the multilayer device, stimulated by a human fingertip.
